# Antidiabetic drug administration prevents bone mineral density loss: Evidence from a two-sample Mendelian randomization study

**DOI:** 10.1371/journal.pone.0300009

**Published:** 2024-03-07

**Authors:** Mingzhu Chen, Shuisen Lin, Wanqiong Chen, Xiaoqiang Chen

**Affiliations:** 1 School of Pharmacy, Quanzhou Medical College, Quanzhou, China; 2 Department of Orthopaedic Surgery, Quanzhou First Hospital Affiliated to Fujian Medical University, Quanzhou, China; Universidade de Trás-os-Montes e Alto Douro: Universidade de Tras-os-Montes e Alto Douro, PORTUGAL

## Abstract

The aim of this study was to investigate the effect of common antidiabetic drugs on BMD by two-sample Mendelian randomization (MR). The single nucleotide polymorphisms that were strongly associated with insulin, metformin, rosiglitazone and gliclazide were extracted as instrumental variables (IVs) for MR analysis. The inverse variance weighted (IVW) method was used as the primary MR method to assess the causal effect of antidiabetic drugs on BMD, and other MR methods, including Weighted median, MR Egger and Weighted mode, were used for complementary analysis. Reliability and stability were assessed by the leave-one-out test. In the present work, IVW estimation of the causal effect of insulin on heel BMD demonstrated that there was a null effect of insulin on heel BMD (β = 0.765; se = 0.971; *P* = 0.430), while metformin treatment had a positive effect on heel BMD (β = 1.414; se = 0.460; *P* = 2.118*10^−3^). The causal relationship between rosiglitazone and heel BMD analysed by IVW suggested that there was a null effect of rosiglitazone on heel BMD (β = -0.526; se = 1.744; *P* = 0.763), but the causal effect of gliclazide on heel BMD evaluated by IVW demonstrated that there was a positive effect of gliclazide on heel BMD (β = 2.671; se = 1.340; *P* = 0.046). In summary, the present work showed that metformin and gliclazide have a role in reducing BMD loss in patients with diabetes and are recommended for BMD loss prevention in diabetes.

## Introduction

Osteoporosis (OP) is a systemic skeletal condition marked by reduced bone density, compromised bone quality and strength, resulting in heightened bone fragility and a greater likelihood of fractures [1]. The typical clinical signs include widespread pain, height reduction, a curved spine, and limited respiratory capacity. These symptoms can significantly impair self-care abilities and life quality [2], especially in middle-aged and elderly individuals, leading to increased mortality rates and healthcare costs. Consequently, OP and fractures related to it have emerged as a critical global health challenge, with their impact potentially equalling that of cardiovascular and cerebrovascular diseases [3, 4]. In an era where societies are increasingly aging, the early prevention and treatment of OP to minimize the incidence of related fractures is an urgent global health priority.

In the wake of accelerated urbanization and rapid economic growth, there has been a notable shift in human lifestyle and dietary patterns, leading to an escalation of diabetes as a pressing global public health concern [5, 6]. This rise in diabetes prevalence is closely linked with a significantly heightened risk of OP and fractures among diabetic individuals, studies reveal that those with type 2 diabetes are 1.7 times more susceptible to hip fractures than their non-diabetic counterparts [7–9]. Such osteoporotic fractures pose a severe threat, especially to the middle-aged and elderly population, and are a leading contributor to mortality and disability in these age groups [10]. The interplay between diabetes mellitus and osteoporosis is complex, often involving hyperglycemia’s adverse effects on bone health and insulin’s role in bone metabolism [10, 11]. There’s an alarming synchrony in the rising trends of both OP and diabetes mellitus [11]. Consequently, for patients grappling with both diabetes and osteoporosis, a dual-focused approach is vital. This involves managing diabetes effectively while simultaneously implementing preventive measures against osteoporosis, aiming to lower patient mortality and alleviate the broader social and economic impacts.

Currently, the therapeutic arsenal for diabetic osteoporosis (DMOP) is diverse, encompassing medications that promote bone synthesis, drugs that inhibit bone metabolism, and various antidiabetic agents [12, 13]. These drugs vary in their mechanisms of action and therapeutic effectiveness. In light of these developments, there’s an increasing need for holistic patient healthcare strategies that not only address the glycemic control in diabetes but also prioritize bone health. Such integrated care approaches are essential for reducing the compounded risks associated with this dual diagnosis, thereby improving patient outcomes and quality of life. This multi-faceted challenge underscores the importance of continued research and development in this field to provide more effective and tailored treatments for those affected by both diabetes and osteoporosis.

In managing diabetic osteoporosis (DMOP), the foremost priority revolves around the effective management of blood sugar levels, the maintenance of proper bone metabolism, and the prevention of bone density deterioration, ultimately contributing to the progression of DMOP [7]. As a result, this research endeavor aims to pioneer a fresh perspective by employing a two-sample Mendelian randomization (MR) approach. This approach seeks to shed light on the potential impact of widely used antidiabetic medications on bone mineral density (BMD). The findings from this study are poised to assist healthcare professionals in making informed decisions when selecting appropriate antidiabetic drugs tailored to the specific needs of various types of diabetic osteoporosis patients.

## Materials and methods

### Study design

In this research endeavor, we undertook an evaluation of the causal impact of antidiabetic medications on BMD utilizing a two-sample Mendelian randomization (MR) approach. We specifically identified four commonly prescribed antidiabetic drugs as the exposures of interest, while BMD served as the primary outcome measure. To facilitate our MR analysis, we extracted Single Nucleotide Polymorphisms (SNPs) as instrumental variables (IVs).

In accordance with the fundamental principles of MR analysis, the chosen IVs had to meet three crucial assumptions. Firstly, they needed to exhibit a robust and significant association with the exposures under investigation, namely, the antidiabetic drugs in our study. Secondly, the selected IVs had to be free from any confounding factors, such as parathyroid hormone/analogues, calcitonin, and estrogen in the context of our study. Lastly, it was imperative that the IVs exerted their effects on the outcome (BMD) solely through their influence on the exposures, rather than through any direct biological mechanisms.

### Dataset sources

All IVs utilized in this study were derived from genome-wide association study (GWAS) summary data. To be more specific, the single nucleotide polymorphisms (SNPs) associated with antidiabetic drugs were sourced from a publicly accessible dataset encompassing European individuals, which had been disseminated by Neale et al. The study included four commonly prescribed antidiabetic medications: insulin, metformin, rosiglitazone, and gliclazide.

In a complementary fashion, genetic variants linked to BMD were acquired from a GWAS executed by Morris et al. [8]. in 2019. This comprehensive investigation examined the genetic determinants of BMD, as measured through heel quantitative ultrasound, across a vast cohort of 426,824 European individuals. Remarkably, this study unveiled 518 genome-wide significant loci (including 301 novel findings), which collectively accounted for a noteworthy 20% of the observed variance in BMD.

### IV extraction and selection

Initially, we selected SNPs that exhibited a strong association with the antidiabetic drug exposures, as evidenced by a stringent significance threshold of *P* < 5*10^−8^. This criterion ensured that the chosen SNPs had a substantial and credible connection to the antidiabetic medications under investigation. Subsequently, we implemented a strategy to eliminate SNPs that were in high linkage disequilibrium (LD) with one another, utilizing criteria based on a threshold of r^2^ = 0.001 and kb = 5000 [14, 15]. This step helped to ensure the independence of our instrumental variables and mitigate potential bias introduced by correlated SNPs. Furthermore, we exercised caution by excluding SNPs that explained more of the variance in the outcome (BMD) than in the exposure (antidiabetic drugs). This was achieved through the Steiger test, which can indicate the presence of reverse causality, suggesting that the outcome may influence the exposure rather than vice versa. To further enhance the reliability of our MR analysis, we implemented the MR-PRESSO method to identify and remove any potential outliers in our instrumental variable set. Outliers can distort MR results and affect the validity of causal inference. Finally, to ensure that IVs were robust and did not introduce weak instrument bias, we computed F-statistics for each SNP. SNPs with F-statistics below the threshold of 10 were deemed weak instrumental variables and subsequently excluded from our analysis. Following this rigorous selection process, the remaining SNPs were deemed suitable instrumental variables and were utilized for our subsequent MR analysis, aimed at assessing the causal relationship between antidiabetic drugs and BMD.

### MR analysis

In the course of this research, we employed a comprehensive array of MR methods to examine the causal relationship between antidiabetic drugs and BMD. Our primary analytical approach was the Inverse Variance Weighted (IVW) method, which is widely recognized as the most commonly used MR analysis. It applies weighted analysis to all SNPs when all SNPs involved are considered IVs. This method seeks to provide robust causal estimates by leveraging the weighted contributions of each SNP [16, 17].

In addition to the IVW method, we conducted supplementary MR analyses using three other methods: the Weighted Median, MR Egger, and Weighted Mode approaches. Each of these methods serves as a valuable complement to our primary analysis. The Weighted Median method imposes a requirement that at least 50% of the SNPs utilized must function as effective IVs. After ranking the included SNPs based on their individual weights, this method derives its analysis result from the median of the corresponding distribution function. It represents a useful approach to ascertain causal effects when a substantial proportion of the instrumental variables are influential. On the other hand, the MR Egger regression analysis offers a unique perspective by estimating the causal effect of antidiabetic drugs on BMD under the assumption of pleiotropy, wherein a single genetic variant may influence multiple traits. The regression slope in MR Egger analysis represents the estimated causal effect. It is worth noting that MR Egger analysis, while informative, often exhibits lower statistical power and yields wider confidence intervals. Consequently, it is commonly utilized as a sensitivity analysis to corroborate and support findings from other MR methods.

By employing this diverse set of MR methodologies, our study aimed to provide a robust and comprehensive assessment of the causal relationship between antidiabetic drugs and BMD, thereby enhancing our understanding of the potential impact of these medications on bone metabolism.

### Statistical analysis

To gauge the consistency and potential sources of variation in our MR analysis, we conducted a Cochran’s Q test, which is commonly employed to assess heterogeneity across the study outcomes. This test helped us identify whether there were significant disparities in the estimated causal effects of antidiabetic drugs on BMD among the individual SNPs used as IVs.

Furthermore, we scrutinized the presence of pleiotropy, a phenomenon where a genetic variant may influence multiple traits, by employing two distinct tests: the MR–Egger intercept test and MR-PRESSO. The MR–Egger intercept test provided insight into the presence of directional pleiotropy, while MR-PRESSO offered a means to detect and mitigate the influence of outliers and pleiotropic SNPs [14].

To ascertain the reliability and robustness of our MR findings, we conducted a leave-one-out test. This procedure involved systematically excluding one SNP at a time from our instrumental variable set and assessing the impact on the estimated causal effects. This sensitivity analysis helped us gauge the stability of our results in the face of potential influential data points.

All of the statistical analyses outlined above were executed using R Studio version 3.6.1. A *P*-value < 0.05 was considered to indicate statistical significance.

## Results

### The causal effect of insulin on heel BMD

We initiated our investigation by examining the potential causal relationship between insulin and heel BMD. In this analysis, we incorporated a total of 7 SNPs as IVs for MR. The IVs set exhibited a commendable average F-statistic, with a mean value of 115.047. Additional details regarding these SNPs can be found in S1 File.

**Fig 1:**
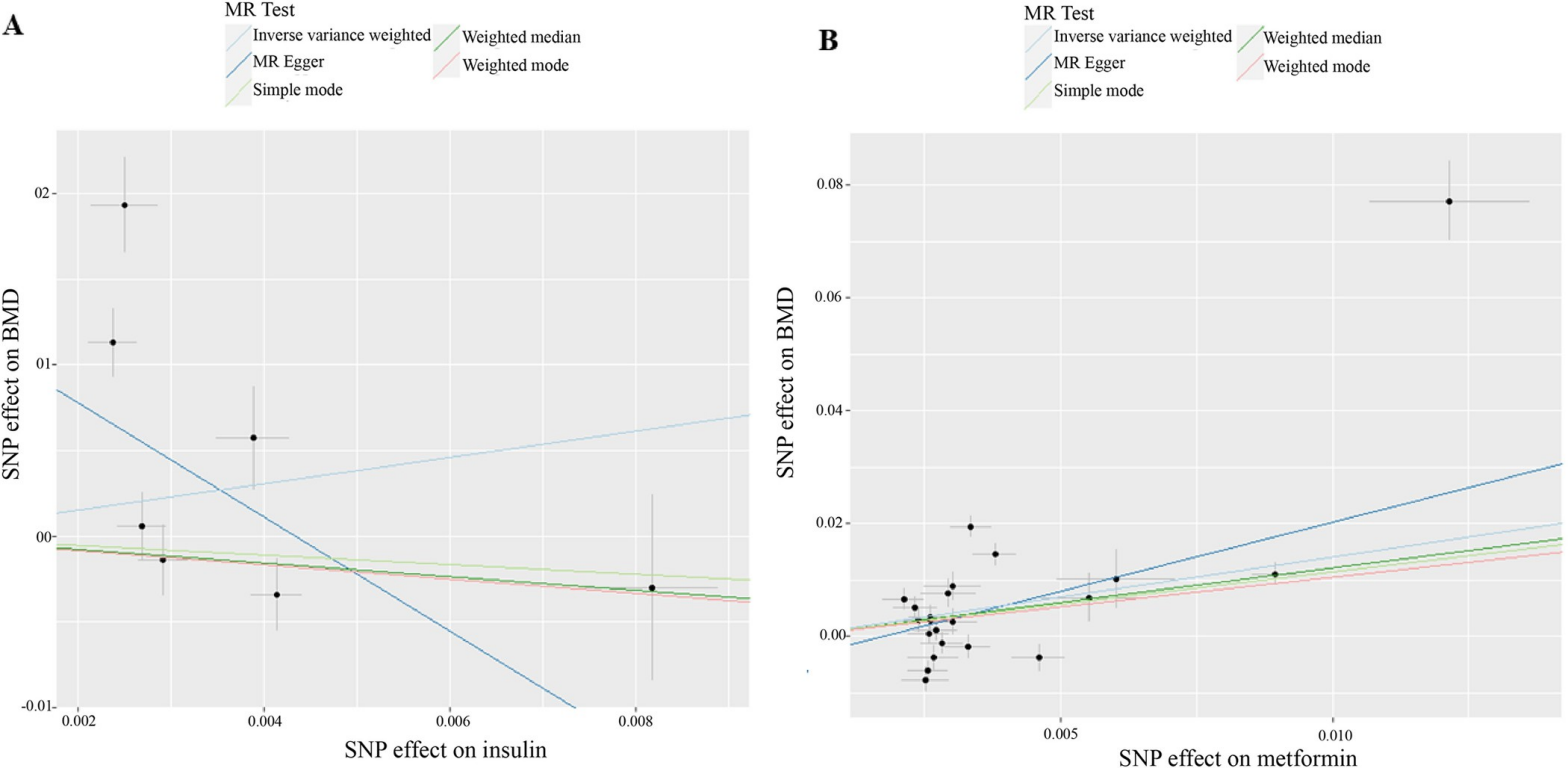
MR analysis of the causal effect of insulin (A) and metformin (B) on heel bone mineral density. MR: Mendelian randomization; SNP: single nucleotide polymorphism; BMD: bone mineral density.

The results of our MR analysis are concisely summarized in Table 1 and visually depicted in Fig 1A. Our primary MR analysis, conducted using the IVW method, indicated a null effect of insulin on heel BMD (β = 0.765; se = 0.971; *P* = 0.430). This outcome suggests that the administration of insulin to individuals with diabetes does not appear to mitigate the loss of BMD.

**Table 1:** The Mendelian randomization analysis results.

Exposures	Methods	Nsnp	β	Se	*P*-value
Insulin	MR Egger	7	-3.327	2.736	0.278
	Weighted median	7	-0.398	0.386	0.303
	Inverse variance weighted	7	0.765	0.971	0.430
	Simple mode	7	-0.277	0.371	0.483
	Weighted mode	7	-0.417	0.359	0.289
Metformin	MR Egger	22	2.446	1.035	2.834*10^−2^
	Weighted median	22	1.224	0.222	3.542*10^−8^
	Inverse variance weighted	22	1.414	0.460	2.118*10^−3^
	Simple mode	22	1.146	0.4891	2.920*10^−2^
	Weighted mode	22	1.053	0.225	1.314*10^−4^
Rosiglitazone	MR Egger	4	0.563	6.218	0.936
	Weighted median	4	-0.858	1.991	0.667
	Inverse variance weighted	4	-0.526	1.744	0.763
	Simple mode	4	-1.002	2.597	0.725
	Weighted mode	4	-0.972	2.619	0.735
Gliclazide	MR Egger	6	2.487	3.371	0.502
	Weighted median	6	2.164	0.562	1.196*10^−4^
	Inverse variance weighted	6	2.671	1.340	0.046
	Simple mode	6	-0.706	1.364	0.627
	Weighted mode	6	2.596	0.584	6.727*10^−3^

Nsnp: the number of single nucleotide polymorphism.

Furthermore, we employed several other MR methods to cross-validate our findings. MR‒Egger, Weighted Median, Simple Mode, and Weighted Mode all yielded similar results, corroborating the absence of a significant causal effect of insulin on heel BMD. Specifically, the estimated effect coefficients (β) and associated P-values for these methods were as follows: MR‒Egger (β = -3.327; se = 2.736; *P* = 0.278), Weighted Median (β = -0.398; se = 0.386; *P* = 0.303), Simple Mode (β = -0.277; se = 0.371; *P* = 0.483), and Weighted Mode (β = -0.417; se = 0.359; *P* = 0.289).

Collectively, these findings consistently point to the conclusion that the use of insulin in diabetic patients does not appear to have a discernible impact on mitigating the loss of BMD.

### The causal association between metformin and heel BMD

Our subsequent investigation delved into the potential causal association between metformin and heel BMD. For this analysis, we meticulously selected a total of 22 SNPs to serve as IVs, detailed information about these SNPs in could be found in S2 File.

The comprehensive outcomes of our MR analysis are thoughtfully presented in Table 1 and visually depicted in Fig 1B. Notably, our primary MR analysis utilizing the IVW method revealed a positive effect of metformin treatment on heel BMD (β = 1.414; se = 0.460; *P* = 2.118*10^−3^). This result suggests that metformin therapy may indeed exert a favorable influence on heel BMD.

To further validate our findings, we employed various other MR methods, all of which consistently supported a positive effect of metformin on heel BMD. The effect coefficients (β) and corresponding P-values for these methods were as follows: MR‒Egger (β = 2.446; se = 1.035; *P* = 2.83410^−2^), Weighted Median (β = 1.224; se = 0.222; *P* = 3.54210^−8^), Simple Mode (β = 1.146; se = 0.4891; *P* = 2.92010^−2^), and Weighted Mode (β = -1.053; se = 0.225; *P* = 1.31410^−4^).

In our quest to ensure the robustness of our findings, we conducted a series of assessments. Cochran’s Q test highlighted the presence of heterogeneity (Q = 272.614; *P* = 1.141*10^−45^), indicating variations in the estimated causal effects among the individual SNPs. However, this heterogeneity did not preclude us from drawing meaningful conclusions, as both the MR–Egger intercept test (MR–Egger intercept = -0.00416; se = 0.00374; *P* = 0.279) and MR-PRESSO detected an absence of pleiotropy, thus affirming the reliability of our results.

**Fig 2:**
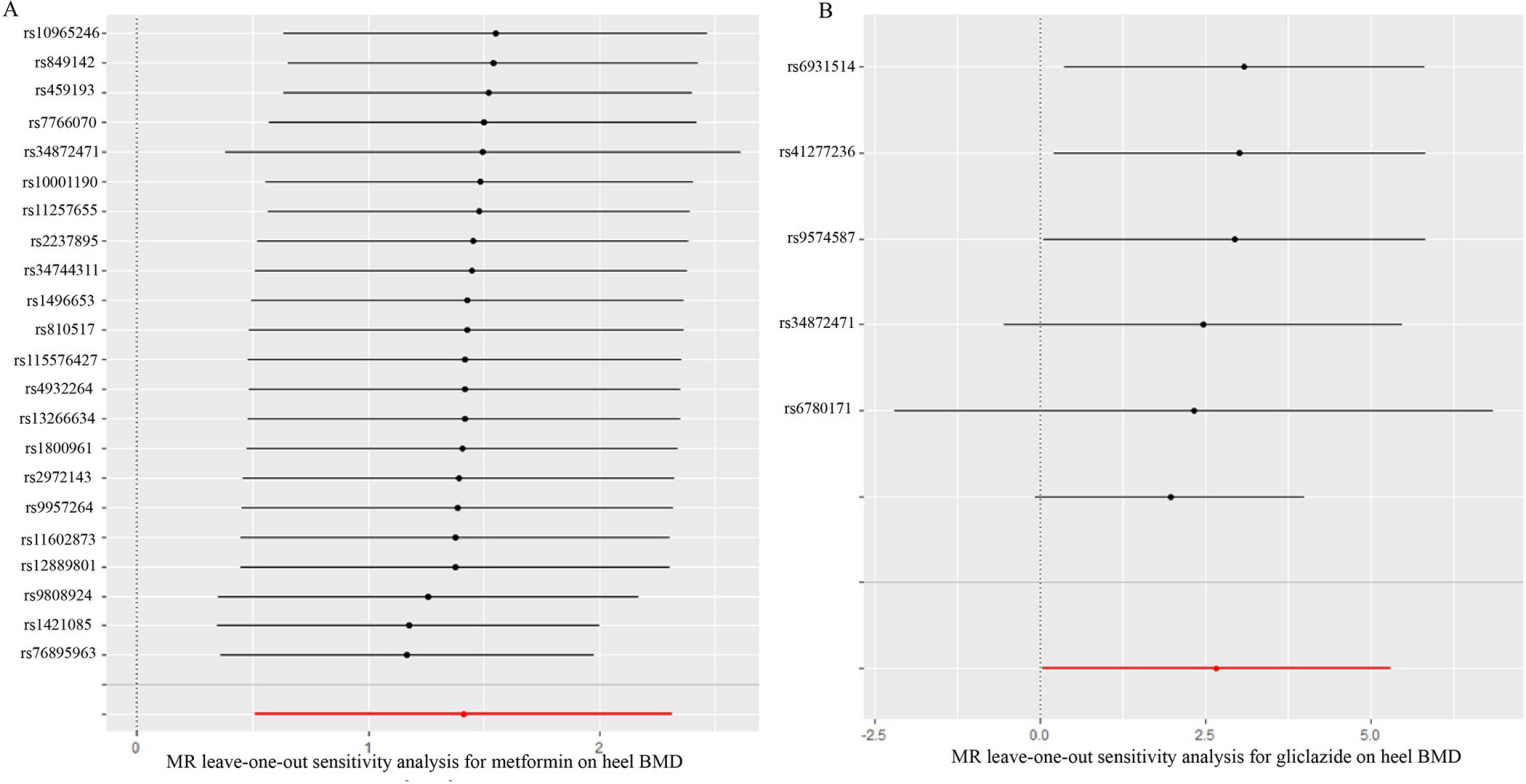
The leave-one-out test results for metformin (A) and gliclazide (B) on heel bone mineral density. MR: Mendelian randomization; BMD: bone mineral density.

Moreover, leave-one-out test demonstrated the stability and dependability of our findings. As illustrated in Fig 2A, the results remained consistent and did not exhibit substantial fluctuations even when individual SNPs were systematically excluded from the analysis. This further bolstered the credibility of our conclusion that metformin treatment may have a beneficial impact on heel BMD.

### The causal relationship between rosiglitazone and heel BMD

In the context of rosiglitazone treatment, we identified a total of 4 relevant Single SNPs associated with this medication. These SNPs were specifically denoted as rs117299843, rs138205523, rs144741037, and rs187455998. Their collective IV set exhibited an average F-statistic of 32.554, indicating their suitability for MR analysis. Detailed information about these SNPs can be found in S3 File.

**Fig 3:**
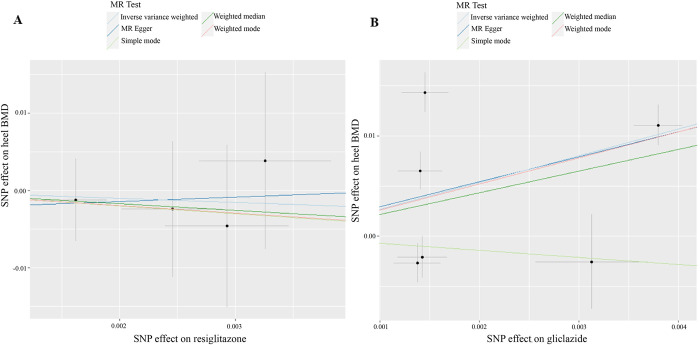
MR analysis of the causal effect of rosiglitazone (A) and gliclazide (B) on heel bone mineral density. MR: Mendelian randomization; SNP: single nucleotide polymorphism; BMD: bone mineral density.

Our MR analysis aimed to elucidate the potential causal relationship between rosiglitazone and heel BMD. The outcomes of this analysis, concisely presented in Table 1 and visually illustrated in Fig 3A, consistently demonstrated a null effect of rosiglitazone on heel BMD.

Specifically, the primary analysis utilizing the IVW method suggested that there was a null effect of rosiglitazone on heel BMD (β = -0.526; se = 1.744; *P* = 0.763) (Table 1 and Fig 3A). This result signifies that rosiglitazone treatment did not appear to have a substantial impact on heel BMD.

To further affirm the absence of a causal relationship, we applied additional MR methods, all of which yielded consistent findings. These methods included MR‒Egger (β = 0.563; se = 6.218; *P* = 0.936), Weighted Median (β = -0.858; se = 1.991; *P* = 0.667), Simple Mode (β = -1.002; se = 2.597; *P* = 0.725), and Weighted Mode (β = -0.972; se = 2.619; *P* = 0.735). Collectively, these results underscore the conclusion that rosiglitazone does not appear to play a significant role in preventing BMD loss in individuals with diabetes.

## The causal effect of gliclazide on heel BMD

Finally, our investigation turned to the assessment of the causal effect of gliclazide on heel BMD. In this particular analysis, we identified a total of 6 SNPs that were deemed suitable IVs for MR study. These SNPs were specifically labeled as rs34872471, rs41277236, rs6780171, rs6931514, rs7925578, and rs9574587. Detailed information concerning these SNPs can be found in S4 File.

The outcomes of MR analysis, which succinctly summarize the results in Fig 3B and Table 1, consistently revealed a positive effect of gliclazide on heel BMD. Our primary MR analysis, conducted using the IVW, demonstrated that there was a positive effect of gliclazide on heel BMD (β = 2.671; se = 1.340; *P* = 0.046). This result suggests that gliclazide treatment may have a favorable influence on heel BMD.

To enhance the robustness of our findings, we employed multiple other MR methods, all of which substantiated the positive relationship between gliclazide and heel BMD. These methods encompassed MR‒Egger (β = 2.4873; se = 3.371; *P* = 0.502), Weighted Median (β = 2.164; se = 0.562; P = 1.19610^−4^), Simple Mode (β = -0.706; se = 1.364; *P* = 0.627), and Weighted Mode (β = 2.596; se = 0.584; *P* = 6.72710^−3^).

In our effort to comprehensively assess the results, we conducted a Cochran’s Q test, which indicated the presence of heterogeneity (Q = 53.905; *P* = 2.192*10^−10^) in the estimated causal effects among the individual SNPs. Nevertheless, the MR–Egger intercept test (MR–Egger intercept = 0.000438; se = 0.00720; *P* = 0.954) and MR-PRESSO consistently pointed to the absence of pleiotropy, ensuring the reliability of our findings. The reliability and stability of our MR analysis were further validated through leave-one-out test, as depicted in Fig 2B. This test demonstrated that our results remained consistent and did not exhibit substantial fluctuations even when individual SNPs were systematically excluded from the analysis.

In conclusion, our comprehensive MR analysis suggests that gliclazide treatment may have a beneficial effect on heel BMD. These findings offer valuable insights into the potential impact of gliclazide on bone health in individuals with diabetes, underscoring its potential role in preserving BMD.

## Discussion

As human societies continue to develop and face the challenges of an aging population, there has been a notable global increase in the incidence of diabetes [18, 19]. This rise is accompanied by a spectrum of acute and chronic complications that pose significant risks to patient health. Notably, among these chronic complications, OP [9] has emerged as a leading cause of persistent, severe pain and functional impairment. This condition is particularly concerning as it markedly increases the likelihood of fractures. Such fractures can lead to high levels of disability, complicating the treatment and rehabilitation process for individuals with diabetes [20, 21]. This intersection of diabetes and osteoporosis presents a complex health issue, necessitating more comprehensive and multidisciplinary approaches to management and care.

In recent years, the intriguing connection between diabetes and OP has captivated the attention of the medical research community. Nevertheless, the intricate mechanisms and elusive causes underlying this complex relationship continue to baffle researchers [7, 10, 13]. A myriad of factors, ranging from genetic predisposition and environmental influences to lifestyle choices, systemic hormonal fluctuations, and local cytokine activity, all contribute in their own distinctive ways to the development of osteoporosis [8, 11, 12, 22]. Consequently, despite concerted efforts, a dependable and safe method for restoring BMD to its normative levels in individuals afflicted with osteoporosis has remained frustratingly elusive.

Compelling evidence now underscores the heightened risk of fractures attributable to osteoporosis among individuals with diabetes [23, 24]. In a comprehensive review conducted by Zhang et al. [25], the impact of various anti-diabetic medications on fracture risk in patients with type 2 diabetes mellitus was meticulously examined. Their findings illuminated that certain drugs, such as voglibose and albiglutide, exhibit a positive effect in reducing fracture risk, while the effects of other pharmaceutical interventions display a spectrum of variability. Consequently, the pressing need to formulate efficacious strategies for the prevention and intervention of osteoporosis in individuals living with diabetes has never been more apparent.

In the current study, we break new ground by employing a pioneering approach, conducting a two-sample MR analysis, to delve into the uncharted territory of investigating the potential roles of commonly used antidiabetic agents in mitigating bone loss. This marks the very first exploration of its kind, shedding light on a novel avenue in our quest to better understand and address the intricate interplay between diabetes and osteoporosis.

The findings from our study have unveiled significant insights into the effects of various diabetes medications on heel BMD. Specifically, our research demonstrates that insulin and rosiglitazone, while used in the treatment of diabetes, do not exhibit the capacity to stave off the reduction in heel BMD. In contrast, both metformin and gliclazide emerge as promising agents, not only in effectively managing blood sugar levels but also in preventing the decline in heel BMD, thereby thwarting the onset of DMOP.

In recent years, metformin has increasingly garnered attention for its potential role in maintaining normal bone mass and regulating bone metabolism. Schwartz et al. [26], in their comprehensive analysis within the Diabetes Prevention Program Outcome Study (DP POS), discovered that prolonged usage of metformin did not yield any significant impact on the BMD of diabetic patients. However, in a study conducted by Wang et al. [27], which involved 120 individuals with type 2 diabetes randomly assigned to either an experimental or control group, the experimental group received high-dose metformin while the control group received a lower dosage. The results from this study indicated that high-dose metformin resulted in a noteworthy improvement in BMD and bone metabolism, aligning closely with the outcomes observed in our own investigation.

Moreover, Araújo et al. [28] undertook research to examine the effects of gliclazide on bone loss using an experimental periodontal disease model. Their study demonstrated that the administration of gliclazide led to a significant reduction in bone loss in rats afflicted with ligature-induced periodontitis. This suggests that gliclazide also plays a vital role in mitigating BMD loss, mirroring the findings of our own study.

Collectively, our research contributes to the growing body of evidence highlighting the potential of metformin and gliclazide in not only effectively managing blood sugar levels but also in safeguarding bone health and averting the development of diabetic osteoporosis. These findings underscore the importance of selecting the right diabetes medications to address not only glucose control but also the overall well-being of individuals living with diabetes.

Previous research has illuminated the underlying mechanisms by which metformin serves as a protective agent against bone loss, with a primary focus on its capacity to promote osteogenic differentiation and mineralization of mesenchymal stem cells [29]. Animal experiments conducted in the past have consistently reported that metformin possesses the capability to enhance and induce osteogenic differentiation in mesenchymal stem cells [30, 31]. Furthermore, in vitro studies have elucidated that metformin can bolster the synthesis of type I collagen, increase alkaline phosphatase activity, encourage extracellular calcium deposition, and stimulate osteocalcin synthesis. Importantly, it has also been observed to facilitate the repair of various cell types in diabetic rats [27]. Beyond these effects, metformin exerts its influence on bone metabolism by impacting the activity of both osteoblasts and osteoclasts. On one hand, it triggers changes in the expression of bone morphogenetic protein and nitric oxide while activating extracellular signal-regulated kinase and AMP-activated protein kinase signaling pathways, thereby influencing the activity of osteoblasts. On the other hand, metformin inhibits osteoclast differentiation and diminishes the activity of the C-terminal propeptide of type I collagen [32]. Similarly, previous investigations have demonstrated that gliclazide has the ability to modulate the production and release of various cytokines, including IL-1β, through the PI3K/AKT signaling pathway. These cytokines, in turn, regulate bone metabolism by influencing the expression of the receptor activator of NF-κB ligand and osteoprotegerin [28, 33]. In essence, these findings underscore the multifaceted roles of metformin and gliclazide in maintaining bone health, offering valuable insights into the intricate molecular mechanisms by which these medications impact bone metabolism and ultimately contribute to the prevention of bone loss in individuals with diabetes.

The present study is subject to certain limitations that warrant consideration. Firstly, the study primarily encompassed a European population, raising questions about the generalizability of these findings to individuals from diverse ancestral backgrounds. Additional research is needed to ascertain the applicability of our results to people of various ethnicities; secondly, an important constraint in our study was the inability to stratify the study population into subgroups based on drug dosage. Consequently, we were unable to conduct a detailed analysis of the protective effects associated with different doses of antidiabetic medications in relation to bone loss. Future investigations should consider exploring the potential dose-dependent effects of these drugs on bone health; lastly, our study exclusively focused on evaluating the protective effects of individual antidiabetic drugs on BMD. However, in clinical practice, the management of diabetes often involves the use of combination therapies comprising multiple antidiabetic medications. Therefore, it becomes imperative to delve into the collective impact of different combinations of antidiabetic drugs on BMD. Further research in this direction is crucial to gain a comprehensive understanding of how various drug combinations influence bone health in diabetic individuals.

## Conclusions

In summary, this study showed that antidiabetic drugs can effectively control blood sugar and at the same time play a role in reducing bone loss, thus reducing the occurrence of DMOP. Metformin and gliclazide are recommended to prevent bone loss in diabetic patients.

## Supporting information

S1 File Details of single nucleotide polymorphisms related to insulin. (CSV)

S2 File Details of single nucleotide polymorphisms related to metformin. (CSV)

S3 File Details of single nucleotide polymorphisms related to rosiglitazone. (CSV)

S4 File Details of single nucleotide polymorphisms related to gliclazide. (CSV)
