# Correction: Antidiabetic drug administration prevents bone mineral density loss: Evidence from a two-sample Mendelian randomization study

**DOI:** 10.1371/journal.pone.0347243

**Published:** 2026-04-14

**Authors:** Mingzhu Chen, Shuisen Lin, Wanqiong Chen, Xiaoqing Chen

The fourth author’s name is spelled incorrectly. The correct name is: Xiaoqing Chen.
